# The MILE study: a motivational, individual and locally anchored exercise intervention among 30–49 year-olds with low levels of cardiorespiratory fitness: a randomised controlled study in primary care

**DOI:** 10.1186/1471-2458-13-1224

**Published:** 2013-12-23

**Authors:** Kirstine Hoj Obling, Kristian Overgaard, Lise Juul, Helle Terkildsen Maindal

**Affiliations:** 1 Department of Public Health, Section for Health Promotion and Health Services, Bartholins Allé 2, 8000 Aarhus C, Denmark; 2 Department of Public Health, Section of Sport Science, Dalgas Avenue 4, 8000 Aarhus C, Denmark

**Keywords:** Maximum oxygen uptake, Cardiorespiratory fitness, Accelerometry, Physical activity, Primary care, Motivation, Social media

## Abstract

**Background:**

Low levels of cardiorespiratory fitness are associated with high risk of non-communicable diseases and all-cause mortality. Physical activity level is the primary determinant of cardiorespiratory fitness in adults. However, knowledge on how to motivate people to engage in physical activity and maintain an active lifestyle is lacking. This study aims to investigate whether a motivational, individual, and locally anchored exercise intervention, in primary care, can improve cardiorespiratory fitness in 30 to 49 year olds with a low or very low cardiorespiratory fitness.

**Methods/Design:**

Two-armed randomised controlled trial with 6 and 12 months follow-up. The primary outcome is cardiorespiratory fitness estimated via a maximal incremental exercise test. Secondary outcomes include physical activity level and sedentary behavior (objectively measured), self-reported physical activity, biochemical parameters (HbA_1C_, HDL- and LDL-cholesterol, and triglyceride), anthropometric parameters and health-related quality of life. A total of 236 participants with low levels of cardiorespiratory fitness classified at a local health check programme will be randomised. The intervention consists of four motivational interviews, a six months membership to a sport club, and a global positioning watch to upload training activity to Endomondo.com. The comparison group will receive standard care: a one hour motivational interview followed by another interview if requested. Effects will be estimated by evaluating the differences in mean changes in cardiorespiratory fitness between the two groups.

**Discussion:**

In new and innovative ways the focus of this study will be to improve cardiorespiratory fitness among a 30–49 year-old at-risk group using social media, Global Positioning System-technology, on-going personal support and individually tailored physical activity.

**Trial registration:**

ClinicalTrials.gov (no.NCT01801956).

## Background

It is well known that low levels of cardiorespiratory fitness are associated with a high risk of noncommunicable diseases and all cause mortality, and that improvements in cardiorespiratory fitness decreases this risk especially among the least fit [1-5]. Although cardiorespiratory fitness has a genetic contribution, physical activity habits are the primary determinant of cardiorespiratory fitness in adults, and changes in physical activity levels result in changes in cardiorespiratory fitness [2,6]. Thus the World Health Organization recommends adults aged between 18–64 to do at least 150 minutes of moderate-intensity aerobic physical activity or at least 75 minutes of vigorous-intensity aerobic physical activity weekly or an equivalent combination of moderate- and vigorous-intensity activity. In Denmark only an estimated 32% fullfill these recommendations [7] and improving physical activity among this group of people is of great importance to public health.

In primary care several simple- and multicomponent physical activity interventions have been conducted in order to increase physical activity. However, the results of these interventions remain inconsistent. In addition, the content of the interventions is often unclearly described, particularly in relation to intensity and fidelity of the intervention delivery and reviews reveal how most of the studies use self-reported measures of physical activity [8,9]. These issues warrant well-described intervention studies using objectively measures of physical activity and specifically designed for, and evaluated in primary care settings.

In Denmark, the “Check your health” programme is aimed at screening all individuals (n ≈ 26,500) in the municipality of Randers aged between 30–49 years for cardiovascular disease during a 5-year period. Identifying people that are insufficiently active and people with a low cardiorespiratory fitness is one of the central objectives of the programme and this is done by self-reported and objective measures of physical activity and cardiorespiratory fitness.

To perform a successful intervention, strategies and techniques to motivate and guide people to adopt healthy choices need to be identified. Some of this may be accomplished by close supervision and personal instruction with healthcare professionals applying communication skills such as motivational interviewing [10,11], which has been found to be an effective approach to changing behaviour. This method specifically offers promise in improvement of cardiovascular health status [12].

Furthermore, a key issue will be to adopt modern technology and social media into the interventions [13]. A Danish report supports this idea and concludes that particularly the 30–50 year olds, an age-group that does not always give high priority to regular physical activity due to lack of time and commitments such as job/further education and establishment of a family, are motivated by the social media and technology [14-16].

Finally, it is also suggested that physical activity interventions should be integrated into the structure of a broader range of community-based organisations e.g. sport clubs in order to offer a wide range of activities, and thereby individualise and anchor the physical activity locally [17].

Using the combination of motivational interviewning, social media, technology and the community organisations may build individual capability and organisational capacity for behaviour change, create new social norms, and promote policy and environmental changes that support higher levels of physical activity across the population in the long-term.

We therefore hypothesise that a locally anchored “primary care package” containing social media and technology, ongoing personal support and individually tailored flexible physical activity will improve cardiorespiratory fitness among 30–49 year-olds with a low cardiorespiratory fitness.

Thus the aims of this paper were to describe:

1. The design of a study evaluating the efficacy of a motivational, individual and locally anchored exercise intervention in primary care aiming to improve cardiorespiratory fitness among 30 to 49 year olds with low levels of cardiorespiratory fitness.

2. The actual intervention to a level of detail that allows its replication.

## Methods

### Study design

**Figure 1:**
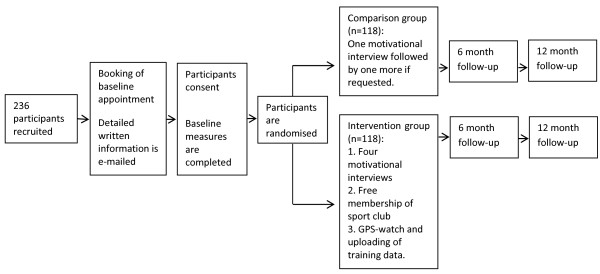
Design and flowchart for the MILE-study.

The study is conducted as a single (researcher) blinded randomised controlled trial higly explanatory in attitude delivered under as optimal conditions as possible [18]. The randomisation sequence will be computer-generated by an independent statistician. Block randomisation will be applied to ensure that couples living together will be randomised to the same group. The allocation to either comparison or intervention group will take place at the front desk at Randers Health Care Centre, and an e-mail will subsequently be sent to either the person in charge of the comparison group or the person in charge of the intervention group (project secretary). See Figure 1 for a flow diagramme of the study.

### Sample size

This study was calculated to detect a 3 ml/kg/minute (sd 6) difference between the intervention- and comparison group at the end of 6 months. The sample size calculation was based on unpuplished data from another randomised controlled health screening programme in Denmark showing a difference in cardiorespiratory fitness of 2,1 (sd 6) over a five year period and previous studies comparing 6 and 24 month intervention effects of a lifestyle physical activity program reporting differences between 3.64 (sd 3,5) and 1.34 (sd 3.37) ml/kg/minute respectively [19]. With a power of 90% using a two-sided p = 0.05 test, a sample size of at least 85 in each group will be necessary. To accommodate non-independence within couples, we used robust variance estimation to obtain valid uncertainty estimates and an extra 6 participants in each group will be added to the sample size. Anticipating a drop-out of 30%, a total of 118 participants will be included in each group.

### Participants

A volunteer sample of 236 participants with low or very low cardiorespiratory fitness expressed as the index of maximum oxygen uptake per minute divided by body weight (ml O_2_/kg/min) will be recruited from the “Check your health” programme to the MILE-study. This corresponds to ≤ 39 and ≤35 ml O_2_/kg/min. for 30–39 and 40–49 year-old men respectively and ≤33 and ≤31 ml O_2_/kg/min. for 30–39 and 40–49 year-old women, respectively [20]. Due to the use of social media and technology in the intervention, the participants must have access to the internet at home. Exclusion criteria will be health problems impeding participation, beta-blocker medication, a blood pressure above 160/100 mmHg, pregnancy, alcoholism, lacking ability to communicate with the staff and plans to move out of the municipality within the year following study start.

At the “Check your health” examination, participants who meet the inclusion criteria will be informed about the MILE-study and receive a pamphlet containing additional information about the study including contact information. Interested participants will be referred directly. If eventually participation is requested, a project secretary can be contacted.

The refered participants will be called and asked questions regarding the in- and exclusion criteria. Allocated participants will be scheduled for baseline measurements in Randers Health Care Centre and detailed information about the content of the study and the study outcomes will be forwarded by mail.

Prior to the baseline test session, a thorough account of the study will be given, study requirements of the participants will be explained and any questions will be answered. If the terms for participation is accepted an informed consent approved by the Ethics Committee, Central Denmark Region, will be signed and baseline measurements started.

### Intervention group

The intervention will be a six month “primary care package” based on motivational, individual and locally anchored physical activity. The content is elaborated in the following:

#### Physical activity

The participants will be requested to follow the National Health Authority’s advice of 30 minutes daily moderate/vigorous physical activity and additional 20 minutes of high intensity physical activity at least twice a week [21].

The participants will receive a six-month free membership for Randers Gymnastic Club, which is a nonprofit local sports club geographically situated in the middle of Randers municipality. Randers Gymnastic Club has existed since 1872, has approximately 8.500 members, and offers a wide range of activities within sport and culture. A total of ten people are permanently employed and other 250 are voluntary certified instructors. Randers Gymnastic Club will coordinate existing activities (Table 1) and implement new activities based on an initial needs assessment analysis in the target group (see section *Needs assessment analysis*). The activities will take place in open classes with other members of the Randers Gymnastic Club and will be delivered by experienced trained instructors. The participants will decide themselves whether they will attend activities in Randers Gymnastic Club, or do moderate/vigorous/high intensity physical activity on their own or in other sport clubs.

**Table 1:** Present activities in randers gymnastic club offered 118 participants in the intervention period

**Activity**	
Forging Cross	Badminton
Power Fit	Spinning intro
Steptoning	Spinning 2
Pilates	Running
Table tennis	Toning/MBL
Swimming	Circuit training
Spinning 1	Walking in the nature
Gymnastics	Zumba fitness

#### Virtual software platform

The participants will be required to upload training activities and pulse data at least once a week to the virtual software platform Endomondo.com via a global positioning system (GPS)-watch (Garmin Forerunner 210). Endomondo.com is an online sports community based on free real-time GPS-tracking of running, cycling, cross-training etc. At this site, it is possible to get a complete training log, to measure training progression and compare data with other study participants or friends. It is also possible to key in activities that are unmeasurable via the GPS-watch eg. water activities. The participants will get to keep the watch if they attend the 12-month follow-up.

#### Motivational interviewing

The intervention group will receive four motivational interviews - at baseline, after three weeks and after three and six months. The purpose of the interviews will be to support and guide the participants to the kind of physical activity that suits them and their everyday life.

The interviews will be based on the five key communication skills used throughout the motivational interview: Asking open questions; Affirming; Reflection; Summarizing and providing information and advice with permission. The guiding principles underlying the motivational interview are [11]:

1. *Engaging* which is the process of establishing a helpful connection and working relationship.

2. *Focusing* which is the process by which a specific direction in the conversation about change is developed and maintained.

3. *Evoking* which involves eliciting the clients’s own motivations for change and lies at the heart of MI.

4. *Planning* which encompasses both developing commitment to change and formulation a concrete plan of action.

The first interview will be a 90-minute long “face to face” interview in Randers Gymnastic Club including introduction to Randers Gymnastic Club, the GPS-watch and uploading of training data http://Endomondo.com.

The two ensuing interviews will be 15-minute telephone interviews including a status on the uploaded activities which the instructors will have access to, and finally a 30-minute face to face interview will be conducted. The motivational interviews will be delivered by trained fitness instructors from Randers Gymnastic Club, who have had a total of 8 hours training on the guiding principles underlying the motivational interview. Guidelines for each interview will be handed out and each interview will be registered. Each exercise mentor will be paid a total of 500 Danish crowns per participant.

### Comparison group

Each participant in the comparison group will be invited to a one-hour motivational interview [11] about their current activity level, motivation and different options for increasing their physical activity level. The interview will be conducted by an experienced internationally certified coach at Randers Health Care Centre, and followed by a second meeting, if requested. This is the current standard care in the municipality.

#### Needs assessment analysis

Before the final planning of the present study, a needs assessment among 100 former “Check your health” participants with a very low VO_2max_ was accomplished. It comprised a questionnaire with questions about the participants’ preferences for activities, transportation to the activities, whether the participants wanted to do activities on their own or in groups, with friends or relatives, what time of the day they preferred activity etc. Results showed that the greatest interests were swimming, running and cycling (Table 2). A total of 61% (n = 61) and 29% (n = 29) were willing to spend 5–15 and 15–30 minutes of transportation, respectively, to and from their activity. Moreover, 42% (n = 42) preferred to do physical activity by themselves and 58% (n = 58) preferred activities in the evening.

**Table 2:** Requested activities among 100 “Check your health” participants before the unset of the Mile-study

**Activity (%)**			
Running	(36)	Zumba	(12)
Swimming	(33)	Spinning	(8)
Cycling	(20)	Weight training	(6)
Fitness	(18)	Soccer	(5)
Badminton	(18)		

Activities requested by less than 5% of the participants: Boxing, dancing, gymnastics, walking, fencing, kayaking, handball, pilates, riding, rowing, squash, Nordic walking, tennis, triathlon, volley and wii.

### Primary outcome

#### Maximum oxygen uptake

Maximum oxygen uptake (VO_2max_) will be measured by a standardised and validated maximal ergometer bicycle test (Monark 939 E Pendulum Ergometer, Monark Exercise AB, Sweden). The bicycle intensity will be commenced at 100 watt (women 75 watt). Every second minute, the load will be increased by 35 watt until the load causes fatigue. The time spent cycling, the highest load achieved and the body weight of the participant will be used to estimate the VO_2max_[22].

The primary outcome will be measured at baseline and 6 and 12 months.

### Secondary outcomes

#### Physical activity level

Physical activity level will be measured by a commercially available accelerometer (Actigraph GT3X-plus) which is one of the most extensively measured validated and accurate devices currently on the market [23]. Studies using accelerometers are often limited to a minimum wear-time of 10 hours/day for 1–4 days/week [24,25]. However to evaluate compliance with recommended weekly amounts of physical activity and due to differences regularly observed between weekday and weekend activities we find it important to monitor activity during all waking hours for one whole week [26]. Thus in the present study the accelerometer will be affixed with an adhesive patch on the right hip at the anterior axillary line of the participant and worn day and night for seven days.

#### Self-reported physical activity

In addition to the objective measures, self-reported physical activity will be measured by questions from the Danish National Health profile questionnaire [27] and a modified version of the question regarding spare-time physical activity used by Bengt Saltin and Gunnar Grimby [28].

#### Biochemical variables

Relevant plasma markers (HbA1c, HDL- and LDL-cholesterol, triglyceride) will be measured by means of a capillary test (DCA Vantage Analyzer, Siemens Healthcare, Siemens AG, Germany, Alere Cholestech LDX System, Alere Denmark).

#### Anthropometric and demographic variables

Arterial blood pressure will be measured in the sitting position (Omron M6 Blood pressure monitor, Omron Healthcare Europe B.V) after resting for at least five minutes. Three consecutive measurements will be obtained and the average of the last two measurements will be used. Body weight, waist circumference and height will be measured to the nearest 0.1 kg and 0.5 cm, respectively. Information regarding age, sex, ethnicity, current smoking status and medication will be obtained from the “Check your Health” database.

#### Health related quality of life

Health-related quality of life will be measured by the SF-12 [29].

Physical activity level and self-reported physical activity will be measured at baseline and 6 and 12 months. The rest of the secondary outcomes at baseline and 12 months.

### Data analysis

The primary analyses will be based on the *intention*-*to*-*treat* principle; however a per-protocol analysis will also be performed. Effect of the intervention on VO_2max_ will be estimated by the difference of the mean changes after 6 and 12 months between the intervention group and the comparison group. Furthermore a subgroup analysis regarding gender will be performed. A Student t-test or nonparametric test will be used for normally and unevenly distributed data, respectively. Loss to follow-up analyses will be conducted via baseline characteristics from Check your health and Danish Registers. Results will be presented with 95% confidence intervals (CI) and p values. P values of <0.05 will be considered as statistically significant.

### Ethical approval

The study will be conducted according to the Helsinki Declaration. It has been registered at ClincalTrials.gov (no. NCT01801956) and approved by the Danish Research Ethics Committee (j.no. 1-10-72-428-12) and the Danish Data Protection Agency (j.no: 2012-41-0183). Recruitment started in the summer of 2013.

## Discussion

Several randomised controlled studies about the promotion of physical activity in primary care have been conducted. To our knowledge however, this will be the first study in primary care that includes both social media, GPS-technology, ongoing personal support and individually tailored physical activity.

In our study, the intervention will be a “primary care package” delivered under as optimal conditions as possible, because we aspire for results that contribute to the understanding of the effect of the intervention under the most ideal circumstances possible. We will use strict inclusion criteria, reproducible intervention components, outcomes that require specific testing, experienced practitioners (trained instructors and a certified coach) to perform both the intervention and the comparison intervention, monitoring of both the participants and the practitioners and follow-up after 6 and 12 months. However, despite this explanatory attitude the “primary care package” will be delivered in a real life setting which may raise some challenges with regard to controlling the intervention, since it will not be possible to control e.g. the timelines and the content of the motivational interviews.

Using strict inclusion criteria is part of the explanatory attitude and a conscious choice. Thus we may include only a selected group of people which may lead to less generalisable results. In addition the participants may be a very motivated group of people which also may be affected by the study activities itself, e.g. the introduction to the study. This may lead to effect dilution due to increased physical activity in any comparison participants.

However, the reproducible intervention makes the intervention implementable in other primary care settings where practitioners can easily be educated as in this study. Using objective measures of the outcomes make the results higly reliable and the nesting in the 5-year programme, will make it possible to monitor a long-term effect of the intervention.

Thus, we find that this study is designed and reported in a way that users of the results can make meaningful judgments about the applicability to their own context. If found to be effective, we therefore believe that the intervention has the potential to be repeated in a more pragmatic design and afterwords sustained and embedded within real world primary care to the benefit of both society and the individual.

## Abbreviations

GPS: Global positioning system.

## Competing interests

The authors declare that they have no competing interests.

## Authors’ contributions

KHO, KO and HTM tailored the intervention and designed the study. LJ contributed to design and analysis considerations. KO drafted the manuscript with all authors providing critical review and final approval.

## Pre-publication history

The pre-publication history for this paper can be accessed here:

http://www.biomedcentral.com/1471-2458/13/1224/prepub
